# Therapeutic effects of probiotics on neonatal jaundice

**DOI:** 10.12669/pjms.315.7921

**Published:** 2015

**Authors:** Wenbin Liu, Huajun Liu, Taisen Wang, Xueqing Tang

**Affiliations:** 1 Wenbin Liu, Department of Pediatrics, Chengdu Military General Hospital, Chengdu 610083, PR China; 2 Huajun Liu, Department of Pediatrics, Chengdu Military General Hospital, Chengdu 610083, PR China; 3 Taisen Wang, Department of Pediatrics, Chengdu Military General Hospital, Chengdu 610083, PR China; 4 Xueqing Tang, Department of Pediatrics, Chengdu Military General Hospital, Chengdu 610083, PR China

**Keywords:** Bifid triple viable, Jaundice, Neonate, Probiotics

## Abstract

**Objective:**

To evaluate the therapeutic effects of probiotics on neonatal jaundice and the safety.

**Methods:**

Sixty-eight neonates with jaundice were divided into a control group and a treatment group (n=34) randomly, and treated by blue light phototherapy and that in combination with probiotics. The serum bilirubin levels were detected before and 1, 4, 7 days after treatment. The time when therapy showed effects and jaundice faded, clinical outcomes as well as adverse reactions were recorded. The categorical data were expressed as (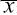
±s) and compared by t test. The numerical data were expressed as (case, %) and compared by χ² test. P<0.05 was considered statistically significant.

**Results:**

Serum bilirubin levels of the two groups were similar before treatment (P>0.05). The levels significantly decreased 1, 4 and 7 days after treatment (P<0.05), but there was no significant inter-group difference on the post-treatment 1st day (P>0.05). The treatment group underwent more significant decreases on the 4th and 7th days than the control group did (P=0.002, 0.001). In the treatment group, the therapy exerted effects on (1.0±0.5) d and jaundice faded on (3.8±1.7) d, which were (2.6±0.6) d and (5.3±2.1) d respectively in the control group (P=0.001, 0.002). The effective rate of the treatment group significantly exceeded that of the control group (P=0.002). There were no obvious adverse reactions in either group.

**Conclusions:**

Probiotics lowered the serum bilirubin levels of neonates with jaundice rapidly, safely and significantly, and accelerated jaundice fading as well. This method is worthy of application in clinical practice.

## INTRODUCTION

In clinical practice, neonatal jaundice is very common, which is mainly manifested as yellowish pigmentation of mucous membranes due to hyperbilirubinemia.^1^ The incidence of neonatal jaundice remains high because of infections, genetic factors and premature birth. If not timely treated, neonates may be subjected to pneumonia or septicemia that severely endangers normal development of intelligence, hearing and the nervous system, or leads to permanent sequelae.^2^

Besides blue light phototherapy, touching and drugs, probiotics have also been used to enhance immunity mainly by regulating bacterial colonies. They can form a biological barrier by specifically binding intestinal epithelial cells through teichoic acid. Therefore, particular attention has been paid to the use of probiotics in treating neonatal jaundice. Therefore, the aim of this study was to evaluate the therapeutic effects of probiotics on neonatal jaundice and the safety, providing clinical evidence for future treatment.

## METHODS

### General Information

Sixty-eight neonates with jaundice who were treated in Chengdu Military General Hospital (Chengdu, China) from July 2012 to August 2014 were selected in this study. This study was approved by the ethics committee of our hospital. Written consent has been obtained from the legal guardians of these neonates.

They were diagnosed according to the criteria for neonatal jaundice in “Zhu Futang Practice of Pediatrics (7th Edition)”^3^ based on the transcutaneous bilirubin levels (>12.9 mg/dL, performed by NJ33A infants tester). Then they were randomly divided into a control group and a treatment group (n=34).

The control group comprised 20 boys and 14 girls with the gestational ages of 38-42 weeks (average: 39.5±0.3). They were aged 5-18.2 d, (11.2±1.3) d in average. Their birth weights ranged from 3.12 to 4.21 kg, (3.75±0.21) kg in average. Causes of jaundice: 10 cases of hemolytic jaundice, 8 cases of infectious jaundice, 8 cases of dysmetabolic jaundice and 8 cases of breast milk jaundice. The treatment group consisted of 22 boys and 12 girls with the gestational ages of 37-41 weeks (average: 39.0±0.4). They were aged 4.2-18.5 d, (11.3±1.2) d in average. Their birth weights ranged from 3.20 to 4.37 kg, (3.86±0.23) kg in average. Causes of jaundice: 8 cases of hemolytic jaundice, 11 cases of infectious jaundice, 8 cases of dysmetabolic jaundice and 7 cases of breast milk jaundice. The two groups had similar age, gender ratio and pretreatment bilirubin levels (P>0.05).

### Treatment Methods

The control group was treated by blue light phototherapy for 3-5 d, 12-24 hour per day. Meanwhile, they were intravenously infused with phenobarbital (Shanghai Sine Pharmaceutical Laboratories Co., Ltd., National Medicine Permit No. H31022038, 30 mg × 100 tablets, batch No. 20130321; dose: 5-8 mg/kg body weight each time, 3 times per day) and 5% albumin (dose: 10-15 ml/kg body weight, once per day).

Based on these, the treatment group was also orally administered with bifid triple viable (Shanghai Sine Pharmaceutical Laboratories Co., Ltd., National Medicine Permit No. S10950032, 0.2 g × 24 s, batch No. 20120524; dose: 2 g/d, completed in three or four times).

### Observation Indices

Fasting venous bloods (3 ml) were drawn before and 1, 4 and 7 days after treatment to detect serum bilirubin levels. After centrifugation at 3000 r/minutes for 10 minutes, the serum was collected and detected by using Mindray BS400 Automatic Biochemical Analyzer with the oxidation method.^4^ The time when therapy showed effects and jaundice faded, clinical outcomes and adverse reactions were recorded. Time of exerting effects: The time of icteric index that was measured with a transcutaneous jaundice meter (JM-103, Chengdu Beisida Instruments Co., Ltd.) decreased.^5^ Clinical outcomes: Markedly effective: Fading of yellowish pigmentation and obvious decrease in serum bilirubin level. Effective: Basic fading of yellowish pigmentation and moderate decrease in serum bilirubin level. Ineffective: Unchanged yellowish pigmentation and maintained or elevated serum bilirubin level.

### Statistical Analysis

All data were analyzed by SPSS18.0. The categorical data were expressed as (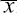
±s) and compared by t test. The numerical data were expressed as (case, %) and compared by χ² test. P<0.05 was considered statistically significant.

## RESULTS

### Serum Bilirubin Levels

Serum bilirubin levels of the two groups were similar before treatment (P>0.05). The levels were significantly decreased 1, 4 and 7 days after treatment (P<0.05), but there was no significant inter-group difference on the post-treatment 1st day (P>0.05). The treatment group underwent more significant decreases on the 4th and 7th days than the control group did (P<0.05) (Table-I).

**Table-I:** Serum bilirubin levels before and after treatment (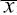
±s, μmol/L).

Group	Before	1 d after	4 d after	7 d after
Control (n=34)	351±48	312±41a	195±40a	108±21a
Treatment (n=34)	347±52	309±42a	155±37a	79±18a
t	0.367	0.876	2.747	5.918
P	>0.05	>0.05	0.002	0.001

Compared with the levels before treatment, aP<0.05.

### Clinical Indices

In the treatment group, the therapy exerted effects on (1.0±0.5) d and jaundice faded on (3.8±1.7) d, which were (2.6±0.6) d and (5.3±2.1) d respectively in the control group (P<0.05). In addition, the two groups had significantly different numbers and times of blue light phototherapy (P<0.05) (Table-II).

**Table-II:** Changes of clinical indices (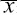
±s).

Group	Time of exerting effect (d)	Time of jaundice fading (d)	No. of blue light phototherapy	Time of blue light phototherapy (h)
Control (n=34)	2.6±0.6	5.3±2.1	2.69±0.96	53.12±19.67
Treatment (n=34)	1.0±0.5	3.8±1.7	2.17±0.76	39.74±9.57
t	3.750	2.121	2.384	3.059
P	0.001	0.002	0.001	0.004

### Clinical Outcomes

The effective rate of the treatment group significantly exceeded that of the control group (P<0.05) (Table-III).

**Table-III:** Clinical outcomes (case).

Group	Markedly effective	Effective	Ineffective	Effective rate (%)
Control (n=34)	18	9	7	79.41
Treatment (n=34)	23	8	3	91.18
χ²				10.65
P				0.002

### Adverse Reactions

Neither group was subjected to obvious adverse reactions, indicating the treatment was safe.

## DISCUSSION

Neonatal jaundice, which is common in clinical practice, can mainly be classified as physiological and pathological ones. The former does not need special treatment,^6^ but the latter, which originates from various factors, easily leads to bilirubin encephalopathy and even brain damage or death. Therefore, it should be timely treated. Mainly manifested as hyperbilirubinemia, neonatal jaundice is caused by enhanced intestinal-hepatic circulation of bilirubin owing to high content and activity of β-glucuronidase (β-GD).^7^ β-GD can hydrolyze the bound bilirubin into unbound one and glucuronide, and the unbound bilirubin boost intestinal-hepatic circulation after being absorbed by intestinal cells.^8^ Recently, intestinal flora, which are diverse and structurally complex, have attracted wide attention as the “Second Human Genome”.^9^

The roles of probiotics in human body have been explained by different pharmacological mechanisms. For example, they can rapidly increase the number of anaerobic bacterial colonies, promote the recovery of intestinal microflora balance, and resist infections in some cases.^10^ On the other hand, intestinal probiotics affect the amount of bilirubin in the enterohepatic circulation by reducing the degradation of bound bilirubin.^11^ Meanwhile, they are able to stimulate intestinal peristalsis, which also benefits the elimination of bilirubin. It has previously been reported that oral administration of probiotics showed markedly better effects than those of routine blue light phototherapy.^12^ Furthermore, the establishment of intestinal probiotic bacteria can perfect the human immune system, thereby affecting the activation and proliferation of sIgA and T lymphocytes as an analogue to active immunity.^13^,^14^ As a result, these exert obvious beneficial effects on neonates.^15^

In this study, neonatal jaundice was treated by using bifid triple viable containing *Lactobacillus bulgaricus*, live *Bifidobacterium* and *Streptococcus thermophilus* that are normal microorganisms in the human intestinal tract.^16^ After being orally taken, they grew in the intestinal tract to produce vitamins and to help the proliferation of normal bacterial colonies. Serum bilirubin levels of the two groups were similar before treatment (P>0.05). The levels were significantly reduced 1, 4 and 7 days after treatment (P<0.05), but there was no significant inter-group difference on the post-treatment 1st day (P>0.05). The treatment group underwent more significant decreases on the 4th and 7th days than the control group did (P<0.05). In the treatment group, the therapy exerted effects on (1.0±0.5) d and jaundice faded on (3.8±1.7) d, which were (2.6±0.6) d and (5.3±2.1) d respectively in the control group, with significant differences (P<0.05). Moreover, the two groups had significantly different numbers and times of blue light phototherapy (P<0.05).

Accordingly, bifid triple viable accelerated jaundice fading by rapidly lowering the bilirubin level, with quick recovery also. Probably, this agent facilitated the growth of normal bacterial colonies in the intestinal tract of neonates, and the resulting metabolites effectively corrected the slightly alkaline environment, thus weakening the activity of β-GD and preventing it from binding and hydrolyzing bilirubin.^17^ Furthermore, the activities of liver enzymes were also enhanced, which benefited the excretion and binding of bilirubin.^18^ The effective rate of the treatment group was significantly higher than that of the control group (P<0.05). Since neither group was subjected to obvious adverse reactions, the treatment was fairly safe.

In summary, probiotics were able to treat neonatal jaundice quickly, safely and significantly, without discernible side effects. Hence, this method is worthy of application in clinical practice.
